# A long journey for acute kidney injury biomarkers

**DOI:** 10.1080/0886022X.2020.1721300

**Published:** 2020-02-12

**Authors:** Dong-Jin Oh

**Affiliations:** Division of Nephrology, Department of Internal Medicine, Myongji Hospital, Hanyang University College of Medicine, Goyang, Korea

**Keywords:** AKI, biomarkers, pre-injury phase, tissue-driven, journey

## Abstract

Acute kidney injury (AKI) is a life-threatening illness that continues to have an in-hospital mortality rate of patients with AKI ranges from 20% to 50% or greater, depending on underlying conditions. However, it has only marginally declined over the past 25 years. Previous authoritative publications have been pointed out that the lack of useful biomarkers for AKI has limited progress in improving the outcomes of this disorder. The purpose of this paper is to review the recent biomarkers involved in the early detection of AKI and main reasons for the failure to identify new AKI biomarkers. So far, several new AKI biomarkers have been discovered and validated to improve early diagnosis, degree of severity, pathophysiology, differential diagnosis, prediction for major kidney adverse events (MAKE, risk groups for progressive renal failure, need for renal replacement therapy [RRT], or death). These biomarkers can be classified into functional, damage and pre-injury phase biomarkers. However, the clinical use of the studied biomarkers in AKI prediction remains unclear because large prospective multicenter trials have failed to demonstrate troponin-like diagnostic performance. Reasons for the failure to identify AKI biomarkers are the heterogeneity of AKI itself, biomarker limitations and long roads to the validation of candidates for new AKI biomarkers. In an effort to overcome these barriers to identifying new AKI biomarkers, kidney biopsy specimens should be obtained and assessed in human AKI populations. Research in this field should be carried out in a pan-social approach rather than conducted by just a few medical institutions.

## Introduction

The in-hospital mortality rate of patients with AKI ranges from 20% to 50% or greater, depending on underlying conditions, and it has only marginally declined over the past 25 years [1]. Previous authoritative publications have been pointed out that the lack of useful biomarkers for AKI has limited progress in improving the outcomes of this disorder [2]. So far, several new AKI biomarkers have been discovered and validated to improve early diagnosis, degree of severity, pathophysiology, differential diagnosis, prediction for major kidney adverse events (MAKE, risk groups for progressive renal failure, need for renal replacement therapy [RRT], or death). However, the clinical use of the studied biomarkers in AKI prediction remains unclear because single or even combined biomarkers provided modest discrimination of AKI [3]. Additionally, while specific therapies have been identified in animal models, their efficacy has not been demonstrated in subsequent human clinical trials, partly because AKI is difficult to identify before loss of organ function has set in, by which time the damage may be irreversible [4]. Therefore, there is growing interest in developing biomarkers that can identify AKI at its earliest stage, when intervention could be more successful. Over the last decade, considerable progress has been made in the discovery and development of new AKI biomarkers. The purpose of this paper is to review the recent biomarkers involved in the early detection of AKI and main reasons for the failure to identifying new AKI biomarkers.

## Traditional biomarkers

### Urine output

Urine output (UO) in AKI can vary from states of oliguria (<500 mL/24 h) to anuria (<100 mL/24 h) to extreme polyuria. While severe AKI can exist despite normal UO, changes in UO can occur long before any biochemical changes are apparent. As such, UO is one of the criteria for defining AKI. UO often decreases before serum creatinine (SCr) concentration increases, making it a more time-sensitive marker of glomerular filtration rate (GFR). However, UO is not nearly as specific for GFR. While it is true that if there is no UO in the absence of obstruction, there can be no GFR, not all reductions in UO signal AKI. Furthermore, while sustained oliguria is invariably associated with AKI, the timeliness of UO as an early indicator is lost in this case.

### Urine dipstick and microscopic examination

Routine dipstick and microscopic examinations of urine are often helpful in determining the cause of AKI. While granular or epithelial casts in urine are increased in acute tubular necrosis (ATN), they lack sufficient sensitivity, specificity, and predictive power for routine clinical use.

### Urinary indices

Diagnostic urinary indices of AKI are the fractional excretion of sodium (FENa), urea (FEurea), uric acid, lithium, urine sodium, and urine osmolality. Reduced effective circulating volume stimulates anti-diuretic hormone (ADH) release, which results in increased distal water and urea reabsorption. Thus, low FEurea (<35%) is more sensitive and specific than FENa in differentiating between prerenal and renal causes of AKI, especially when diuretics have been administered. FEurea and FENa have low diagnostic sensitivity in distinguishing azotemia associated with renal vasoconstriction and intact tubular function from established AKI with tubular dysfunction.

### Tubular enzymes

Traditional urinary biomarkers, including low- and high-molecular-weight proteins (α_1_-microglobulin, β_2_-microglobulin, retinol-binding proteins, etc.), brush border antigens (Na^+^/H^+^ exchanger isoform-3), urinary enzymes (α-glutathione S-transferase, γ-glutamyl transpeptidase, N-acetyl-β-D-glucosaminidase, etc.), and Tamm-Horsfall protein, have not entered clinical routine for AKI patients due to the lack of sufficient validation, lack of standardized assays, and changes in the specificity of the patterns of urinary marker excretion with advancing renal dysfunction.

## Functional biomarkers

### Serum creatinine

Currently, the standard diagnostic tools for AKI detection are SCr concentration and UO, both of which are markers of renal function, but not kidney injury [5]. SCr is an integrator of various intra- and extra-renal functions, and its concentration indicates the balance between creatinine generation and excretion. Unfortunately, due to the non-linear relationship between GFR and sCr, the increase of creatinine may be within the reference range in early phase of AKI and may overlooked. SCr does not indicate the severity of the injury until a steady state has been reached in AKI. Therefore, SCr is a delayed and insensitive biomarker of changes in kidney function; moreover, its concentration does not differentiate between structural kidney damage and functional hemodynamic triggers, and it could be affected by many factors. Especially in intensive care unit patients, creatinine-based definitions of AKI can be influenced by baseline creatinine estimation, fluid overload, malnourishment and muscle wasting. Future investigations should address the estimation of true SCr values and the use of renal functional reserve (RFR) as an important supportive tool for screening and prediction of kidney function recovery or progression to CKD.

### Cystatin-C

Cystatin-C is produced at a constant rate by nucleated cells, filtered by the glomerular cells, almost completely reabsorbed and catabolized (but not secreted) in the proximal tubule. Over the past decade, Cystatin C (CyC), a serum measure of renal function, is a stronger predictor of the risk of death and cardiovascular events in elderly persons than is creatinine [6]. Koyner *et al* demonstrated that urinary CyC was superior to SCr and plasma CyC in the early diagnosis of acute kidney injury following adult cardiac surgery [7]. Roos *et al* performed a systematic review comparing the diagnostic accuracy of plasma and urine CyC with SCr in patients undergoing cardiac surgery. They concluded that the diagnostic accuracy for impaired renal function favors urine CyC [8]. However, while Cys-C levels are a more precise indicator of kidney function than SCr concentration, they seem to be influenced by old age, large doses of corticosteroids, hyperthyroidism, inflammation, neoplasia, smoking and alcohol consumption [9]. Currently, it is unclear whether the value of Cys-C is generalizable to all forms of AKI or specific to particular populations.

## Damage biomarkers

### Neutrophil gelatinase-associated lipocalin

Neutrophil gelatinase-associated lipocalin (NGAL) is a widely expressed 25 kDa protein of the lipocalin family. After ischemic or nephrotoxic kidney injury, intra-renal NGAL is dramatically up-regulated at the transcript and protein levels [10]. Elevated NGAL is detectable in the urine as early as 3 h after injury, and *in vivo* data have suggested the thick ascending limb and the collecting duct as the sites of intra-renal NGAL production, although proximal tubule cells secrete NGAL *in vitro* in response to ATP depletion [11]. Plasma NGAL also increases in AKI as a result of increased hepatic production, and NGAL is filtered by the glomerulus and taken up by the proximal tubules in a megalin-dependent manner [12]. A prospective study of pediatric patients undergoing cardio-pulmonary bypass (CPB) for corrective cardiac surgery found urinary NGAL to be a powerful early marker of AKI [receiver operating characteristics (ROC) level >0.99], preceding any increase in SCr concentration by 1–3 days [13].

### Kidney injury molecule-1

Kidney injury molecule-1 (KIM-1) is a 38.7 kDa trans-membrane protein that contains extracellular mucin and immunoglobulin domains. The basal expression of KIM-1 is low in the normal kidney. However, it is up-regulated after ischemia-reperfusion injury (IRI), and it can be localized to proliferating de-differentiated epithelial cells of the proximal tubule 48 h after injury [14]. Several reports have shown that KIM-1 appears to be a highly sensitive indicator of AKI in non-cardiac surgical patients as well as after cardiac surgery [15,16]. Han *et al.* demonstrated that increased KIM-1 levels were associated with a greater than 12-fold risk for the presence of ATN [17]. Furthermore, Liangos *et al.* studied urinary KIM-1 and NAG in 201 patients with establishedAKI and found that elevated levels of urinary KIM-1 and NAG were significantly associated with the clinical composite endpoint of death or dialysis requirement, even after adjustment for disease severity or co-morbidity [18].

### Interleukin18

Interleukin18 (IL-18) is a 22 kDa pro-inflammatory cytokine that increases in the kidney after IRI in a caspase-1-dependent manner [19]. Patients with ATN had significantly greater median urinary IL-18 concentrations than those with other conditions (healthy controls, patients with pre-renal azotemia, urinary tract infection, CKD, and nephrotic syndrome) [20]. Median urinary IL-18 concentrations measured in the first 24 h after kidney transplantation were higher in patients who received a cadaveric kidney that developed delayed graft function compared to patients who received a cadaveric kidney with prompt graft function and patients who received a kidney with prompt graft function from a living donor [20]. However, the performance of urinary IL-18 measured 1 day before AKI was modest (ROC = 0.73) [20].

### Liver-type fatty acid-binding protein

Liver-type fatty acid-binding protein (L-FABP) is a 14 kDa protein from the large super- family of lipid-binding proteins. The 14-kD L-FABP protein can be localized predominantly in the proximal tubule [21,22]. Noiri *et al* demonstrated that increase of urinary L-FABP was observed at 1 h after ischemia, even in the 5-min IRI group and urinary L-FABP was superior to BUN and urinary NAG for early and accurate detection of acute tubular necrosis in different models of animal AKI [23]. Susantitaphong *et al.* conducted a meta-analysis of diagnostic test studies assessing the performance of urinary L-FABP in AKI, identifying the estimated sensitivity of urinary L-FABP level for the diagnosis of AKI was 74.5% and specificity was 77.6% [24]. Subsequently, Ho *et al* performed systematic review and meta-analysis in adult patients having cardiac surgery, reporting the urine biomarkers NGAL (16 studies), KIM-1 (6 studies), and L-FABP (6 studies) exhibited composite AUROCs of 0.69 to 0.72 in the immediate 24-h postoperative period [25]. Although urinary L-FABP may be a promising biomarker for early detection of AKI and prediction of dialysis requirement and in-hospital mortality, previous papers suggested that potential value of L-FABP needed to be validated in large studies and across a broader spectrum of clinical settings.

## Initial success of damage biomarkers in specific clinical settings

Most early AKI biomarker validation studies have been performed on patients after CPB in that acute renal dysfunction occurs in high up to 40% of adults after cardiac surgery and the timing of the kidney injury is well-known and pathophysiological mechanisms are apparant [13,26–28] or on patients with renal transplantation [29] in the same sense. Acute renal failure (ARF) complicates up to 10% of cardiac surgical procedures in infants and children with congenital heart disease [30]. In addition, these children are unique in that co-morbidities such as advanced age, CKD, diabetes, chronic inflammatory diseases, atherosclerotic disease are usually absent, making them an ideal group for investigation of biomarkers as predictors of early ischemic renal injury [13]. Therefore, many early trials assessed AKI biomarkers in children. An initial prospective human study of urinary NGAL was performed in the pediatric CPB context [13]. Subsequently, multiple studies have confirmed the predictive ability of NGAL in the pediatric CPB context [31,32]. In addition to NGAL, IL-18, KIM-1, and LFABP have been studied in the post-CPB setting [33].

## Failure of the TRIBE-AKI study

The Translational Research Investigating Biomarker Endpoints in AKI (TRIBE-AKI) study evaluated the performance of urinary γ-glutamyl transpeptidase ( γ-GTP), alkaline phosphatase, NGAL, Cys-C, KIM-1, and IL-18 in AKI diagnosis. Upon entry to the ICU, no biomarker had an ROC above 0.7 in the diagnosis or prediction of AKI [34]. Similarly, the initial post-operative levels of urine IL-18, urine NGAL, and plasma NGAL performed only modestly for the outcome of mild AKI, and urine NGAL was not independently associated with AKI when considering the clinical variables after cardiac surgery in adults. The results of four novel urine biomarkers and one plasma biomarker (urine NGAL, IL-18, KIM-1, and L-FABP, and plasma NGAL) were provided. Of all the novel biomarkers, only urine IL-18 and plasma NGAL in adults were helpful in predicting progression of AKI when measured on the day of clinical AKI diagnosis. Combinations of three biomarkers in adults from two different time points and combinations of two biomarkers in children from two time points were found to be able to increase the ROC for AKI up to 0.78 [32]. In summary, the clinical use of single damage biomarkers in AKI prediction remains unclear because large prospective multicenter trials, such as the TRIBE-AKI study (*n* = 1530), have failed to demonstrate troponin-like diagnostic performance [3,34].

## Various clinical scenarios of AKI based on function, damage, and stress

**Figure 1.**
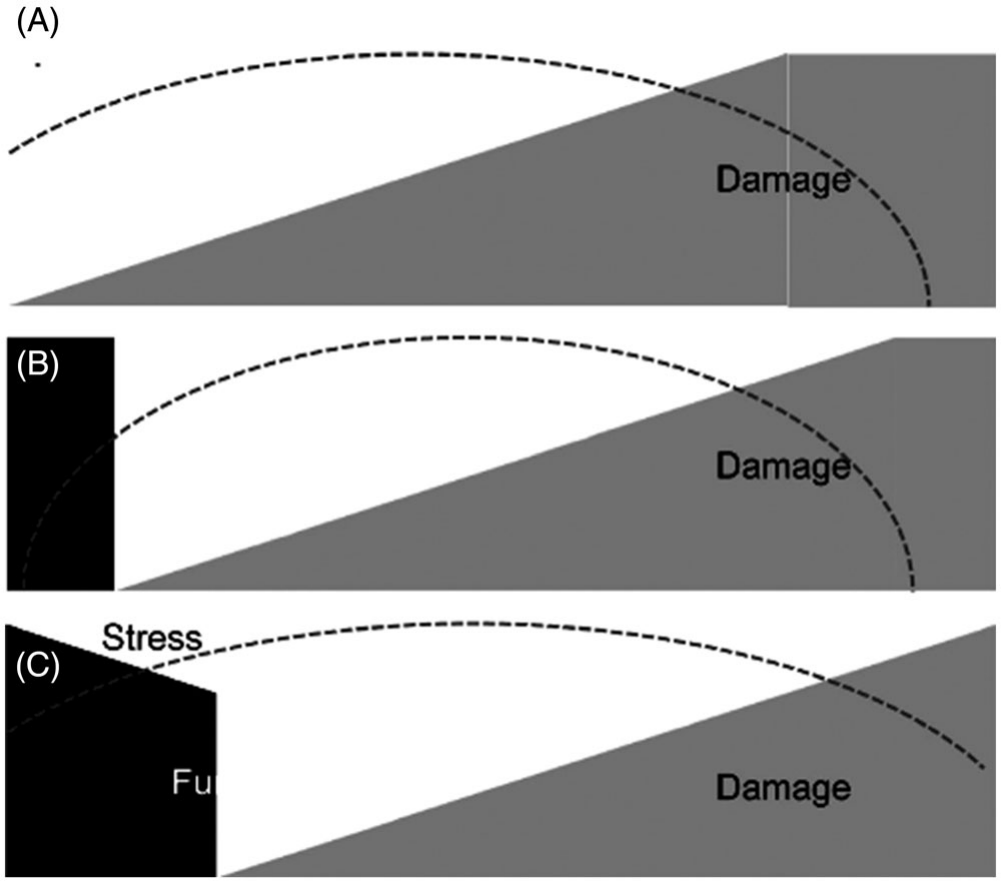
Various clinical scenarios of AKI based on function, damage, and stress. Adapted from Kellum (2015) [35].

Over the last decade, a number of novel damage biomarkers have been evaluated for their capacity to detect kidney damage and predict the development of AKI. However, the relationship between decreasing function and increasing damage is not as straightforward as might be assumed (Figure 1). The characteristic pattern whereby damage precedes loss of function (panel A) may be seen in some cases of AKI and affords an opportunity to detect “subclinical” AKI before function start to fall. Unfortunately, other patterns might also occur. For example, functional decline may start to occur alongside damage (panel B), and in some cases function may start to decline even before damage (panel C), although this is more likely scenario in CKD but not so much in AKI. This makes damage markers difficult to use for predicting AKI. However, other markers might actually measure the “stress” occurring at the cellular level before damage or loss of function [35].

## Paradigm shift to pre-injury phase biomarkers

**Figure 2.**
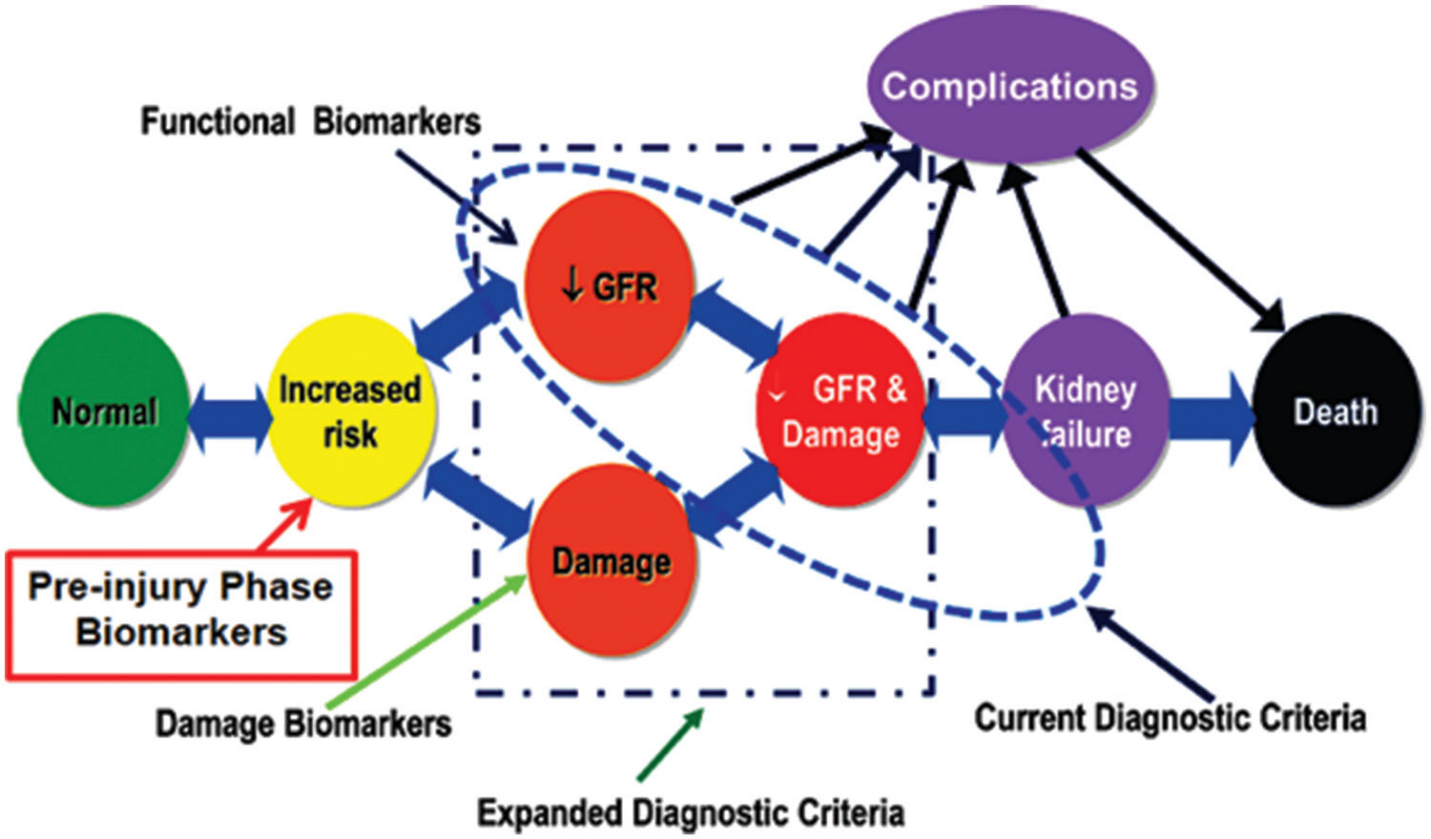
Paradigm shift to pre-injury phase biomarkers. Modified from Murray (2014) [38].

Katz *et al* proposed that the pre-injury phase that leads to AKI can be described as “acute kidney stress” (AKS) after the report of the identification of cell cycle arrest biomarkers that signal the potential development of AKI is part of an evolution in the molecular diagnosis and understanding of AKI [36,37]. These biomarkers are released by kidney cells along a path which may lead to AKI during a pre-injury phase. AKS is defined as the increased-risk phase (pre-injury phase) that leads to AKI [38]. This “AKS” may or may not lead to damage and functional decline. But it is the earliest possible the process of AKI can be detected. Consequently, it might be time to modify the conceptual framework of the early diagnosis of AKI (Figure 2) [38].

## Pre-injury phase biomarkers

### Stress biomarkers

**Figure 3.**
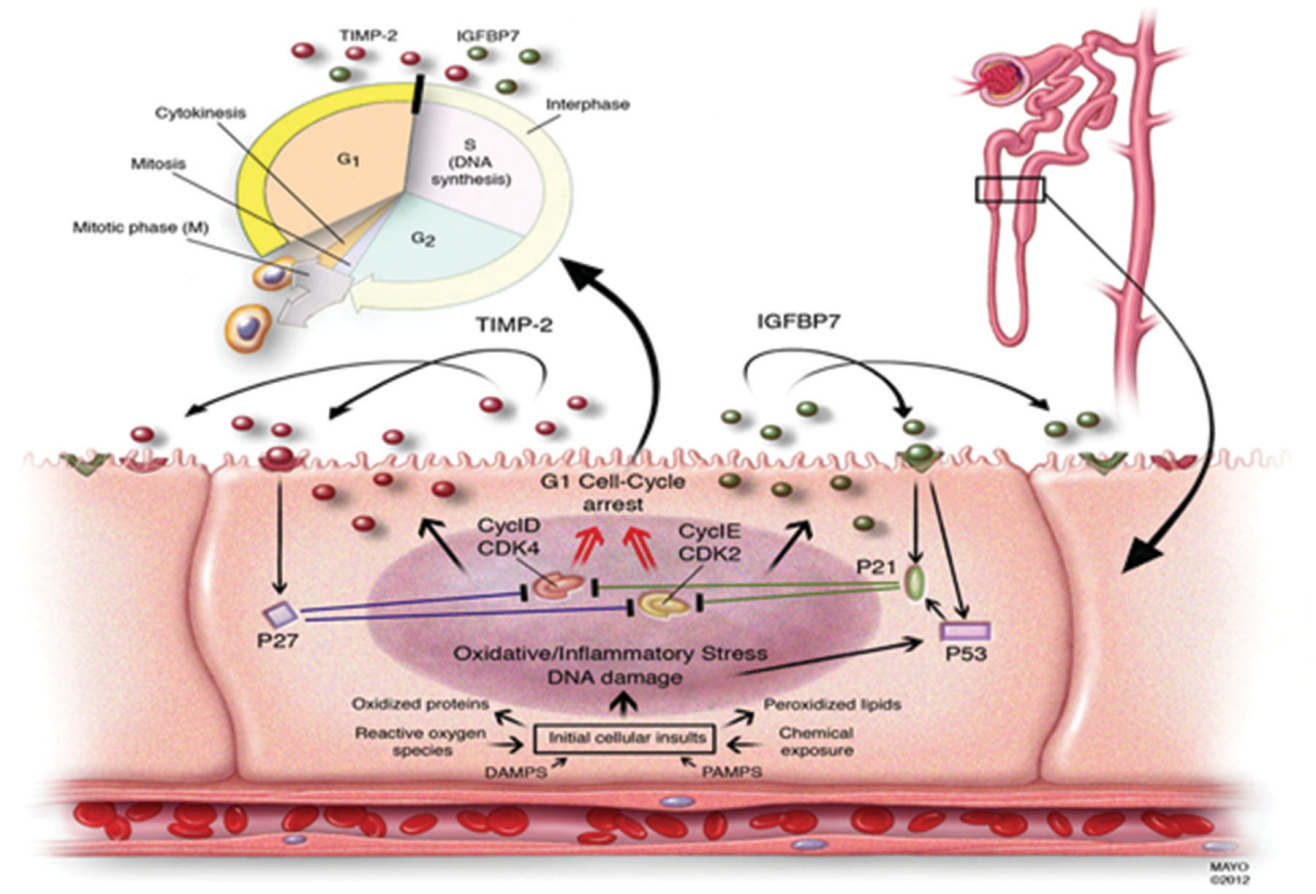
Proposed mechanistic involvement of stress biomarkers in AKI. This figure was originally published under a Creative Commons Attribution 4.0 International License (https://creativecommons.org/licenses/by/4.0/) as part of the following article: Kashani, K., Al-Khafaji, A., Ardiles, T. et al. Discovery and validation of cell cycle arrest biomarkers in human acute kidney injury. Crit Care 17, R25 (2013). https://doi.org/10.1186/cc12503 [37].

In 2013, Kashani *et al.* reported the results of a prospective international observational investigation (the Sapphire study) of tissue inhibitor of metalloproteinases-2 (TIMP-2) and insulin-like growth factor-binding protein 7 (IGFBP7) in a heterogeneous group of critically ill patients (Figure 3) [37]. IGFBP7 and TIMP-2, were validated with an ROC of 0.80 together (0.76 and 0.79 on their own, respectively). [TIMP-2]•[IGFBP7] was significantly superior to all previously described markers of AKI (*p* < 0.002), including NGAL and KIM-1, none of which achieved an area under the curve (AUC) greater than 0.72. Next, the Opal and Topaz study showed the cut-off value and risk assessment using the urinary [TIMP-2] •[IGFBP7] [39,40]. The urine [IGFBP7]*[TIMP2] biomarker is already FDA approved and commercially available in the United States (NephroCheck^®^). However, the summary of the FDA’s evaluation clarifies that [TIMP-2]•[IGFBP7] is not a standalone test. False-positive results are common, and they will be magnified if the test is used inappropriately in low-risk patients. In addition, this biomarker should not be used in ambulatory practices, and it is not beneficial in patients with established KDIGO stage 2 or 3 AKI. The cost benefit of the [TIMP-2]•[IGFBP7] test is unknown. Currently, there are no data to support the premise that early recognition of kidney injury with the [TIMP-2]•[IGFBP7] test or other AKI biomarkers prevents the progression of AKI or is associated with a cost-benefit to the patient or institution [41].

### microRNAs

microRNAs (miRNAs) are endogenous single-stranded non-coding mRNAs of about 19-23 nucleotides. It is estimated that miRNAs regulate more than half of the protein-coding genes in humans [42]. In the past years, researchers have begun to investigate the relevance of miRNAs to AKI, with many miRNAs being implicated. Some of them contribute to pathogenesis by regulating apoptosis and inflammation, amplifying or reducing acute injury responses, while others regulate fibrosis and angiogenesis, participating in renal recovery or progression to fibrosis. The biological and pathological functions of many miRNAs in AKI are still not fully understood. Certain miRNAs have been investigated for their potential as novel biomarkers for the early detection or prognostication of AKI. Given the complex pathophysiology and dynamic nature of AKI, a miRNA panel may be more feasible as a predictor than a single miRNA [43]. Further validation studies are needed to evaluate the clinical utility of such a panel.

### Development-related molecules

Wnt/β-catenin signaling in the adult kidney is relatively silenced, although it has an essential role in kidney development [42–44]. Over the past several years, studies have demonstrated that Wnt/β-catenin signaling could play a pivotal role in promoting tubular repair and regeneration after AKI induced by either IRI or nephrotoxins [45]. The Dickkopf (DKK) family of proteins (DKK1–4) are expressed in the developing kidney, but shut off in adult life. They have been shown to disrupt Wnt binding to its co-receptors and inhibit β-catenin activation. DKK3 is a stress-induced, exclusively proximal tubular epithelia-secreted controller of the Wnt/β-catenin pathway, so its concentration in urine may serve as a biomarker of the pre-injury phase of kidney injury [46]. They may be associated with the type of kidney injury and potentially the outcome [47,48]. Therefore, they are not needed for the diagnosis of late stage AKI but may be an overstatemet to indicate that these have no role in KDIGO stage 2 and 3 AKI. Further validation studies are needed to evaluate the clinical utility of such development-related molecules.

## Other promising biomarkers

### Hemojuvelin

Hemojuvelin (HJV), a glycophosphatidylinositol (GPI)-linked membrane protein, is highly expressed in liver and skeletal muscles. The molecular weight of HJV is 42 kDa for the soluble form (sHJV) [49], and can be passed through glomerular filtration and reabsorbed by the renal tubules [50] Increased iron content in the kidney and urine is observed in human and animal models of AKI [51], and increased iron load can induce renal tubular cell injury [52]. There is evidence that the increased expression of the hemojuvelin-hepcidin ferroportin pathway is an intrinsic response to iron overload conditions during AKI. Therefore, urine HJV (uHJV) has the potential to be an early AKI biomarker in response to iron homeostasis during AKI, which may explain the temporal relationship between uHJV and its predictive capacity [53].

Table 1 is a suggested classification of mostly available biomarkers to date.

**Table 1.** Suggested classification of mostly available biomarkers to date.

Functional biomarkers	Tubular enzymes	Damage biomarkers	Pre-injury phase biomarkers
Creatinine (S)	AAP (U)	KIM-1 (U)	[TIMP-2]•[IGFBP7] (U)
Cystatin-C (S)	AP (U)	NGAL (S, U)	MicroRNAs (U)
	α-GST (U)	IL-18 (U)	Wnt (S, U)
	γ-GTP (U)	L-FABP (U)	DKK (U)
	NAG (U)	Hemojuvelin (U)	
		Clusterin (U) CYR-61 (U) CystatinC (U) Albumin (U)	
		Osteopontin (S, U) Cytochrome-C (U) Epidermal growth factor (U) Malondialdehyde (U)	

Abbreviations; S, serum; U, urine; AAP, alanine aminopeptidase; AP, alkaline phosphatase; α-GST, α-glutathione-S-transferase; γ-GTP, γ-glutamyl transpeptidase; NAG, N-acetyl-β-glucosaminidase; KIM-1, kidney injury molecule-1; NGAL, Neutrophil gelatinase-associated lipocalin; IL-18, interleukin-18; L-FABP, Liver-type fatty acid-binding protein; CYR, cysteine-rich protein; TIMP-2, tissue inhibitor of metalloproteinases-2; IGFBP7, insulin-like growth factor-binding protein 7; Wnt, wingless-related integration site; DKK, Dickkopf.

### Osteopontin – emerging role in septic AKI

Osteopontin (OPN) is an extracellular matrix protein involved in the inflammatory response: as an integrin-binding protein, it modulates leukocyte activation, migration and differentiation as well as cytokine secretion both in acute and chronic inflammation [54–58]. It has been shown that OPN circulating levels not only are elevated in sepsis [58], but they also progressively increase throughout Systemic Inflammatory Response Syndrome (SIRS), sepsis and septic shock [59] and they are associated with higher mortality rates both in animal models [60] and septic patients [59,61]. Recently, Castello *et al*. reported that OPN plasma concentration was found to be an independent predictor of sepsis and the diagnostic receiver operating characteristic (ROC) curve resulted in an area under the curve (AUC) of 0.878 and plasma OPN levels were positively correlated to plasma creatinine. However, this relation was not explained by the development of AKI, since no difference was found in OPN concentration between AKI and non-AKI patients [62]. Therefore, further study should be necessary to elucidate the role of OPN in septic AKI.

## Summaries of biomarkers’ role in current status

In spite of the above negative descriptions, biomarkers in both blood and urine have been studied extensively in the research setting and are on the verge of clinical practice to improve diagnosis of AKI. Biomarkers can be localized to specific areas or functions within the nephron. Recently, Parikh *et al*. suggested that biomarkers such as IL-18, NGAL, LFABP, KIM-1 can help to characterize glomerular or tubular function; glomerular, tubular, or interstitial injury; inflammation; or repair [63]. Further, biomarkers can improve diagnosis of AKI in various clinical settings including acute interstitial nephritis, acute tubular injury, hepatorenal syndrome, and cardiorenal syndrome. Finally, Reese *et al*. suggested that in delineating which donor AKI kidneys will have poor versus favorable outcomes in recipients, urine NGAL and L-FABP provided modest incremental value in predicting worse recipient 6-month eGFR, especially in those with DGF [64]. On the contrary, repair phase protein known as YKL-40 was associated with less DGF and better long-term eGFR [64]. They suggested the proposed paradigms aiming to enhance the clinical diagnosis, management, and prognosis of AKI through the combined use of clinical markers and novel inflammatory, injury, and repair biomarkers, even adding of repurposing existing biomarkers such as urine microscopy score, urine albumin, FENa. Despite biomarkers’ usefulness, there are not many medical institutions that can be easily measured on-site and the cost-effectiveness aspect should be overcome. To date, independent of the diagnostic accuracy of any biomarker for AKI, in clinical practice the early detection of AKI has no impact on clinical decisions. Moreover, therapeutic possibilities for AKI are very limited and the benefit of an early or late start of renal replacement therapy is not clear. Therefore, the early or late detection of AKI in most cases does not have any influence on the clinical course of the patient. This partially may explain why most biomarkers have not been included in the panel of classic laboratory parameters used in the clinical setting. More so, the use of biomarkers without proper clinical risk stratification will yield to suboptimal biomarker performance. To overcome this challenge, the renal angina index was established to risk-stratify patients with AKI on the basis of the severity of the clinical setting and the percentage change in creatinine clearance.

## Reasons for the long journey to identifying AKI biomarkers

### The heterogeneity of AKI itself

**Figure 4.**
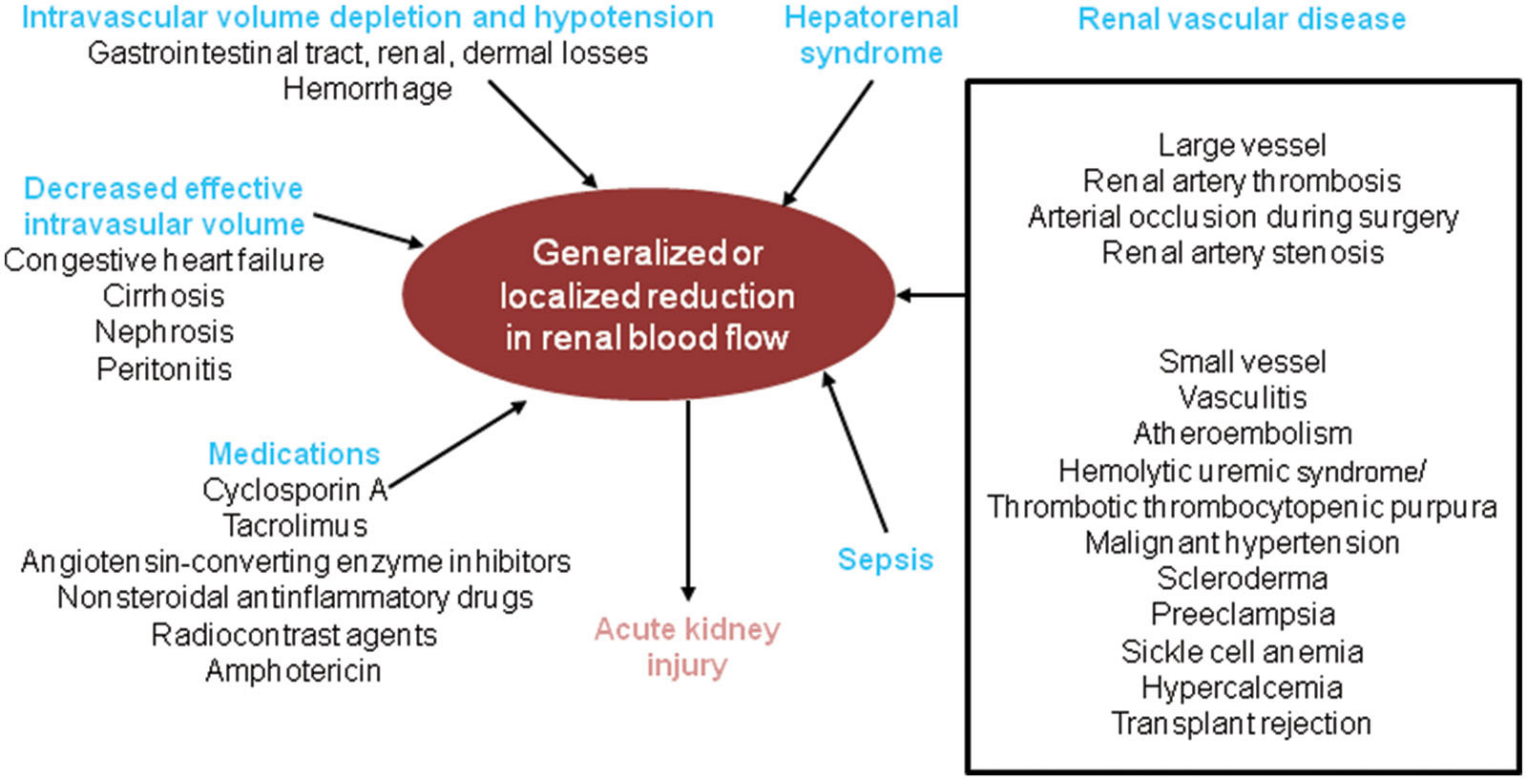
Causes of reduction in generalized or regional renal blood flow (RBF) adapted from Bonventre (2011) [65].

Despite numerous clinical trials, AKI remains a cause of significant morbidity and mortality for which there is no effective intervention. Barriers to translating the successes in animal studies to efficacy in human clinical trials include the heterogeneity of the patient population with AKI with respect to the underlying etiology of renal injury and patient co-morbidities, the complexity of the pathogenesis of AKI, such as hemodynamic or inflammatory status, genetic background, use of nephrotoxic compounds, and the late timing of the enrollment and initiation of experimental interventions (Figure 4) [65]. Unfortunately, this heterogeneity of AKI subtypes is a significant limitation for large population studies in human subjects. In this context, the use of classic clinical predictive models associated with novel renal biomarkers (both biological and genetic) may be the only way to refine the methods of treatment and improve the prognosis for patients.

### Biomarker limitations

Biomarkers have some important limitations that should be known. Unlike troponin in acute coronary syndrome (ACS), none of the reported biomarkers are entirely specific for AKI. NGAL may be elevated in the settings of sepsis, CKD, UTI and the lack of specific cut-off values [66–68]. KIM-1 may be elevated in the settings of chronic proteinuria and inflammatory diseases [69,70]. L-FABP may be strongly associated with anemia in nondiabetic patients [71]. IL-18 has no certain prediction of AKI in adults [69]. [TIMP-2]x[IGFBP7] may be elevated in the setting of diabetes [72]. After AKI occurs, biomarker levels remain elevated for a period of time. This makes assessment of AKI timing rather challenging [73].

### A long road to the validation of candidates for new AKI biomarkers

**Figure 5.**
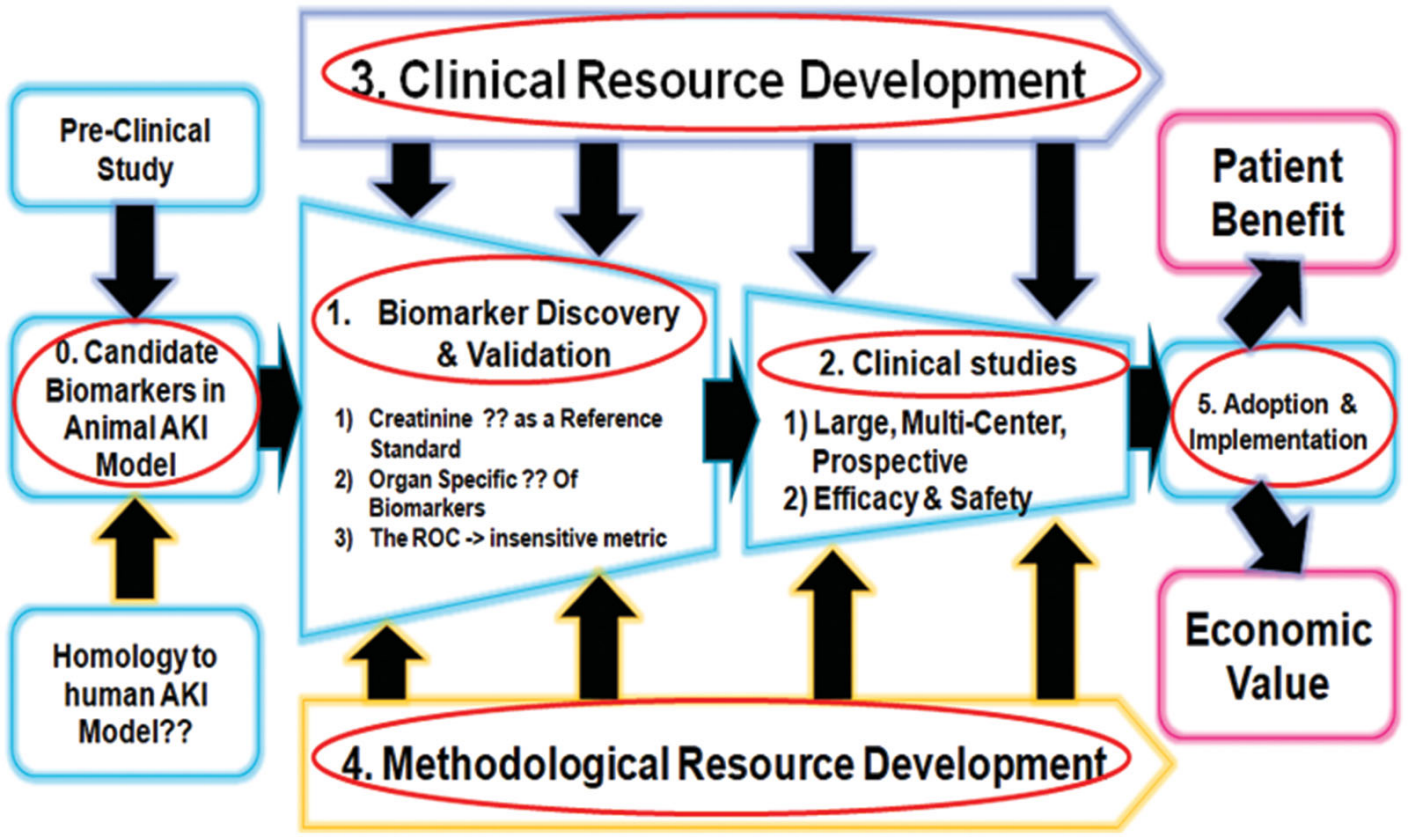
The reasons of the long road to the validation of candidates for new AKI biomarkers [75].

Desirable characteristics of clinically applicable AKI biomarkers include: (1) noninvasive and easy to perform at the bedside or in a standard clinical laboratory using easily accessible samples such as blood or urine, (2) rapidly and reliably measurable using a standardized assay platform, (3) highly sensitive to facilitate early detection and with a wide dynamic range and cut-off values that allow for risk stratification, (4) highly specific for AKI and enable the identification of AKI subtypes and etiologies, and (5) strong biomarker properties on receiver-operating characteristic (ROC) curves [74]. Urine represents an ideal body fluid for AKI biomarker assessment as it can be obtained noninvasively and repeatedly from a spontaneously voided sample or from an indwelling bladder catheter. Nevertheless, there is a difference between biomarkers that can be freely filtered in the glomerulus thus any increase in their plasma concentration (due to damage to extra renal tissues) may result in high urinary concentration (thus losing specificity) and high molecular weight markers that are not freely filtered and are thus more specific when measured in the urine. The reasons of the long road to the validation of candidates for new AKI biomarkers are as below; (1) Candidates for new biomarkers are found in animal AKI models, but these models are not homologous to the human AKI condition. (2) During biomarker discovery and validation, SCr concentration is not sufficient as a reference standard, no kidney-specific biomarkers have been identified, and the ROC is an insensitive metric. (3) Large multi-center prospective studies take a long time. (4) Simultaneously, standardized clinical and methodological resource development should be established. (5) When new biomarkers are adopted and implemented, they must feature patient benefit and economic value (Figure 5) [75].

## The important role of noble biomarkers in AKI prediction models

As AKI tends to occur in patients with common risk factors and in certain medical setting, risk prediction models for AKI have been developed. Conventional AKI risk prediction model development is performed with variable selection, construction of model, testing the discrimination and calibration, and an additional validation study. Novel biomarkers, such as NGAL, TIMP-2, IGFBP7, KIM-1, hemojuvelin, osteopontin, associated with the risk of AKI have been reported in previous studies [53,76–78]. Especially, Kashani *et al* tested the predictive value of novel urine biomarkers, TIMP-2 and IGFBP7, with ICU patients, and suggested a clinical model to predict the risk of AKI [37]. Although the study had limitations regarding AKI risk prediction, as their main goal was to incorporate new urinary markers as risk factors, the study showed that the inclusion of novel biomarkers could improve the robustness of a prediction model in early periods of critical care. Although there is no consensus regarding using such biomarkers to predict AKI, several have shown promising results [37,53,78–80]. Therefore, a novel biomarker that directly reflects kidney injury may further improve prediction in the future. Considering several biomarkers have been reported to directly be released from kidney injuries, such as NGAL or KIM-1, measuring those biomarkers in the early phase may further provide methods to promptly predict an AKI event. Although AKI risk prediction models including novel biomarkers may not be commonly applicable in general hospitals, such studies may help circumvent the limitation of the creatinine-based definition of AKI and facilitate the availability of such biomarkers. Further efforts to develop AKI prediction models based on comprehensive clinical data, artificial intelligence, improved delivery of care, and novel biomarkers may help improve patient outcomes through precise AKI risk prediction [81].

## Conclusions

 Over the past thirty years, despite an increasing amount of literature on the performance of various biomarkers in clinical studies, their full potential has not been realized when compared with the confining indicator of SCr, and information is limited on how these biomarkers could be used by clinicians to manage patients with AKI. And, based on the above comments, it has been found that there are limitations to the application of biomarkers obtained from AKI animal models that are quite different to AKI in humans. In an effort to overcome the long journey to identifying new AKI biomarkers, studies for AKI biomarkers should be classified into ischemic, nephrotoxic, septic, co-morbidity-related (acute cardiorenal syndrome, etc) AKI. In addition, kidney biopsy specimens should be obtained and assessed in human AKI populations to create a kidney tissue pathology and move toward tissue-driven definitions of AKI. Finally, it is believed that this area of research should be carried out in a pan-social approach rather than conducted by just a few medical institutions. To find biomarkers, which have excellent performance in early diagnosis, risk assessment, treatment response and prognosis of AKI, will be the first step to overcoming devastating AKI.
